# The impact of a brief mindfulness meditation intervention on cognitive control and error-related performance monitoring

**DOI:** 10.3389/fnhum.2013.00308

**Published:** 2013-07-09

**Authors:** Michael J. Larson, Patrick R. Steffen, Mark Primosch

**Affiliations:** ^1^Department of Psychology, Brigham Young UniversityProvo, UT, USA; ^2^Neuroscience Center, Brigham Young UniversityProvo, UT, USA

**Keywords:** mindfulness, meditation, event-related potential, error negativity, error-related negativity, post-error positivity, error positivity, cognitive control

## Abstract

Meditation is associated with positive health behaviors and improved cognitive control. One mechanism for the relationship between meditation and cognitive control is changes in activity of the anterior cingulate cortex-mediated neural pathways. The error-related negativity (ERN) and error positivity (Pe) components of the scalp-recorded event-related potential (ERP) represent cingulate-mediated functions of performance monitoring that may be modulated by mindfulness meditation. We utilized a flanker task, an experimental design, and a brief mindfulness intervention in a sample of 55 healthy non-meditators (*n* = 28 randomly assigned to the mindfulness group and *n* = 27 randomly assigned to the control group) to examine autonomic nervous system functions as measured by blood pressure and indices of cognitive control as measured by response times, error rates, post-error slowing, and the ERN and Pe components of the ERP. Systolic blood pressure significantly differentiated groups following the mindfulness intervention and following the flanker task. There were non-significant differences between the mindfulness and control groups for response times, post-error slowing, and error rates on the flanker task. Amplitude and latency of the ERN did not differ between groups; however, amplitude of the Pe was significantly smaller in individuals in the mindfulness group than in the control group. Findings suggest that a brief mindfulness intervention is associated with reduced autonomic arousal and decreased amplitude of the Pe, an ERP associated with error awareness, attention, and motivational salience, but does not alter amplitude of the ERN or behavioral performance. Implications for brief mindfulness interventions and state vs. trait affect theories of the ERN are discussed. Future research examining graded levels of mindfulness and tracking error awareness will clarify relationship between mindfulness and performance monitoring.

## Introduction

The practice of meditation and its effect on cognition and health is receiving increased attention in the mainstream science literature. A number of studies show that meditation practice enhances cognitive function and can alter neural pathways (e.g., Cahn and Polich, 2006; Jha et al., 2007; Tang et al., 2007; Zeidan et al., 2010; Xue et al., 2011; Tang et al., 2012; Teper and Inzlicht, 2013). Mindfulness meditation involves bringing one's complete attention to the experiences occurring in the present moment, in a non-judgmental and accepting way. This cultivation of conscious attention and awareness by the regular practice of mindfulness meditation is related to increased ability to focus attention, to changes in regional cerebral blood flow and white matter connectivity in areas such as the anterior cingulate cortex (ACC) and the dorsolateral prefrontal cortex (dlPFC), and to changes in electroencephalogram (EEG) and event-related potentials (ERPs; Cahn and Polich, 2006; Manna et al., 2010; Xue et al., 2011; Yu et al., 2011; Froeliger et al., 2012; Brown et al., 2013; Teper and Inzlicht, 2013). Several studies of neural changes associated with meditation practices using EEG have shown consistent alterations in alpha and theta band activity (Lagopoulos et al., 2009; Baijal and Srinivasan, 2010). Furthermore, ERP studies have shown meditation is associated with increased attentional acuity among meditators (see Cahn and Polich, 2006 for review). In conjunction with EEG and ERP performance, improvements in attention and self-regulatory processes through meditation interventions have been linked to more efficient cardiovascular functioning (Tang et al., 2009). The pathways through which mindfulness leads to physiological changes and improvement in health and the length of meditation needed to alter physiological and cognitive processes, however, have not been extensively studied. The purpose of the current study was to examine the influence of brief mindfulness meditation on systolic and diastolic blood pressure and the neural correlates of cognitive control and error-related performance monitoring.

A growing body of research links meditation to improved attention and cognitive control. Cognitive control refers to the ability to govern thoughts and actions in accord with internal intentions and involves a complex interplay between dlPFC and ACC-mediated neural mechanisms (Botvinick et al., 2001; Miller and Cohen, 2001). Long-term expert meditators show increased attention abilities and altered MRI during meditative practice vs. control conditions (Cahn and Polich, 2006; Brefczynski-Lewis et al., 2007; Holzel et al., 2007; Moore and Malinowski, 2009; Kozasa et al., 2012; Moore et al., 2012). Similarly, experienced mindfulness meditators performed better on a Stroop interference task and a concentration and endurance test, including electrophysiological indices of attention, indicating stronger attention abilities for practiced meditators relative to non-meditators (Moore and Malinowski, 2009; Moore et al., 2012). Novice meditators also show improved performance and greater efficiency on the Stroop task after engaging in meditation exercises (Wenk-Sormaz, 2005; Chan and Woollacott, 2007). Studies involving functional MRI show that the ACC and prefrontal cortex (including dorsolateral and medial PFC) are significantly affected by meditation, with experienced meditators showing improved efficiency and response inhibition relating to these areas (Allen et al., 2012; Froeliger et al., 2012; Kozasa et al., 2012).

Improvements in attention and cognitive control abilities are not limited to meditators who are adept. Brief meditation practices with novices have produced significant changes in attention abilities and higher-order cognitive processes relative to pre-meditation baselines or non-meditators (Jha et al., 2007; Tang et al., 2007, 2009; Chambers et al., 2008; Zeidan et al., 2010; Moore et al., 2012). For example, Moore et al. (2012) employed a 16-week meditation program, 10-min-practice daily, in their experimental group and observed improved focus attention and efficiency of cognitive resources. Jha et al. (2007) examined an 8-week mindfulness program with improved attention and Tang et al. (2007) had students meditate daily for 20 min for 5 days and found improved attention and self-regulation relative to a relaxation control group. This group of researchers also found increased ACC activity and processing efficiency associated with meditation practice in novices (Tang et al., 2007, 2009, 2012).

One function that relies primarily on ACC-related neural processes is monitoring performance for conflict and errors. An electrophysiological index of performance monitoring is the error-related negativity (ERN). The ERN is a negative deflection in the scalp-recorded ERP that occurs within 100 ms after the commission of an error and is generated by the ACC (Falkenstein et al., 2000; van Veen and Carter, 2002; Stemmer et al., 2004; Brazdil et al., 2005; Roger et al., 2010). Multiple theories about the functional significance of the ERN report different possibilities ranging from the detection of competing response stimuli, to a reinforcement learning signal, to an affective response to mistakes (see van Veen and Carter, 2002; Olvet and Hajcak, 2008; Hoffman and Falkenstein, 2012). Other studies, meanwhile, strongly indicate that ERN amplitude is influenced by affective processes, such as emotional distress or negative affect (e.g., Luu et al., 2000; Vidal et al., 2000; Hajcak et al., 2004; Larson et al., 2006; Inzlicht and Al-Khindi, 2012). Taken together, there remains ambiguity about the functional significance of the ERN, but there is a relationship with conflict and emotional processes.

A second component of the ERP associated with error-related performance monitoring is the post-error positivity or error positivity (Pe). The Pe is a more posterior component than the ERN and tends to occur between approximately 100 and 400 ms after error commission. There are several theories regarding the functional significance of the Pe. A growing body of evidence suggests that the Pe is associated with the conscious processing and awareness of errors and performance abilities (Nieuwenhuis et al., 2001; Endrass et al., 2007, 2012; Larson and Perlstein, 2009; Shalgi et al., 2009; Hughes and Yeung, 2011). Additional studies of the Pe suggest that it is similar to the P300 (specifically the P3b) in that it is reliably larger in amplitude when there is increased motivational significance or salience of the error (Overbeek et al., 2005; Ullsperger et al., 2010). Taken together, current data suggest the Pe is associated with post-error processing that is modulated by the level of attention/arousal and motivational significance the participant places on the mistake (Overbeek et al., 2005; O'Connell et al., 2007; Ullsperger et al., 2010; Endrass et al., 2012). Source localization studies suggest the cingulate cortex and potentially the insula are contributors to the generation of the Pe, although the localization and topography of the Pe is more variable than that for the ERN, with some studies showing it is either more anterior or more posterior than that for the ERN (e.g., Herrmann et al., 2004; Overbeek et al., 2005; O'Connell et al., 2007; Vocat et al., 2008; Ullsperger et al., 2010).

In the first study of error-related performance monitoring and mindfulness meditation, Teper and Inzlicht (2013) showed that expert meditators have increased ERN amplitude relative to controls that was related to measures of emotional acceptance. They interpreted this difference to be present largely as the result of a propensity toward greater emotional acceptance in those engaging in mindfulness meditation. There were no differences between non-meditators and meditators for Pe amplitude; however, the control participants appeared to show slightly more positive Pe amplitude than did the mindfulness participants. Thus, it appears that the increased emotional acceptance associated with mindfulness mediation may increase error-related performance monitoring, but not necessarily conscious awareness and salience of errors.

If there are emotion-based changes in error-related performance monitoring, it would be beneficial to understand if these effects are only present in long-term meditators or if short-term meditation affects these same error-related processes. Interestingly, a recent study by Ramsburg and Youmans (in press) found that a brief 15-min mindfulness exercise, compared to a resting control group, resulted in improved academic performance. The authors suggested that changes in attention might have accounted for the observed differences. We still know little about the pathways through which mindfulness leads to physiological changes and improvement in health—including the relationship between the ACC, autonomic functioning, and mindfulness. The current study can help further our understanding in these areas.

Meditation appears to impact general physiological functioning via pathways connecting the ACC and the autonomic nervous system (Tang et al., 2009). Blood pressure, a key indicator of autonomic function, is significantly lowered by meditation and meditation interventions have shown blood pressure reductions similar to those obtained with diet and exercise interventions (see Anderson et al., 2008 for meta-analytic review). For example, an 8-week meditation intervention (practicing 20 min a day) led to decreased blood pressure with effects lasting at a 1-year follow-up (Carlson et al., 2007). Short-term meditation interventions have also been shown to reduce blood pressure. In a 2-week meditation study where participants practiced meditation and yoga for 2 h per day, blood pressure was significantly lower at the end of the intervention (Ankad et al., 2011). Taken together, meditation interventions performed by expert and novice meditators seem to produce consistent desirable results regarding autonomic functioning and cardiovascular health, with blood pressure being a consistent indicator of these changes, and can potentially be used as an indicator of whether a brief mindfulness intervention is effective in inducing a calm and mindful state.

In the current study, we tested the hypotheses that a brief mindfulness intervention would reduce blood pressure and improve the cognitive control function of performance monitoring in novice meditators. Specifically, consistent with Teper and Inzlicht (2013), we predicted increased amplitude ERN in those assigned to the mindfulness condition relative to the control participants. We did not have specific hypotheses about Pe amplitude. We also examined post-error slowing as an index of cognitive control function (see Danielmeier and Ullsperger, 2011 for review). Given previous findings showing increased post-error slowing is associated with larger amplitude ERN and increased cognitive control (e.g., Danielmeier and Ullsperger, 2011; Wessel and Ullsperger, 2011) and Teper and Inzlicht's (2013) finding of increased ERN amplitude in individuals who practice mindfulness meditation, we expected increased post-error slowing in the mindfulness group vs. the control group. We used systolic and diastolic blood pressure as an indicator of the impact of the meditation intervention. We expected decreased systolic blood pressure across the mindfulness intervention in the mindfulness group relative to the control participants. This study is novel in that it is not known if brief mindfulness meditation practice in a sample of novice meditators will affect performance monitoring and cognitive control functions.

## Materials and methods

### Participants

The Institutional Review Board at Brigham Young University approved all study procedures and the authors do not declare any conflicts of interest. Sixty-two individuals were recruited from undergraduate psychology courses and randomly assigned to either a mindfulness group or a control group (initial *n* = 31 per group). Data from seven participants, three from the mindfulness group and four from the control group, were excluded; one due to equipment malfunction and six due to having fewer than six useable error trials after artifact rejection and correction (Olvet and Hajcak, 2009; Larson et al., 2010). Thus, final study enrollment included 28 individuals (12 female) in the mindfulness group and 27 (14 female) in the control group. Groups did not differ in sex distribution, χ^2^(1) = 0.45, *p* = 0.50. Demographic information of the final sample as a function of group is provided in Table 1. There were no group differences in age, depressive symptoms as measured by the Beck Depression Inventory—Second Edition (Beck, 1996), or state- or trait-anxiety levels as measured by the State-Trait Anxiety Inventory (Spielberger et al., 1983). Exclusion criteria were assessed via participant report and included previous practice in mindfulness meditation, current or previous diagnosis of a psychiatric disorder, current substance abuse or dependence, neurological disorders, head injury with loss of consciousness, left-handedness, or uncorrected visual impairment. Thus, all participants were neurologically- and psychiatrically-healthy individuals unpracticed in mindfulness meditation.

**Table 1:** **Descriptive and performance information for mindfulness and control participants**.

	**Mindfulness(*n* = 28)**		**Control (*n* = 27)**		**Analysis**	
	**Mean**	***SD***	**Mean**	***SD***	***t***	***p***
Age (years)	19.9	2.0	20.6	2.3	−1.3	0.18
BDI-II score	6.7	5.7	8.9	9.6	−1.0	0.34
STAI-State	30.1	8.5	31.1	8.2	−0.4	0.66
STAI-Trait	32.0	8.4	36.1	9.9	−1.6	0.11
Congruent-trial RT (ms)	375.9	26.1	378.4	39.3	−0.3	0.78
Incongruent-trial RT (ms)	429.4	24.2	428.5	38.9	0.1	0.92
Post-correct RT (ms)	410.2	23.4	409.9	39.1	0.0	0.97
Post-error RT (ms)	420.5	28.5	421.0	38.5	−0.1	0.95
Congruent-trial error rates (%)	2.4	2.0	3.0	3.9	−0.7	0.52
Incongruent-trial error rates (%)	10.2	6.1	10.0	6.5	0.1	0.91
Number of correct trials in ERPs	629.7	126.7	654.4	145.4	−0.7	0.50
Number of error trials in ERPs	42.2	29.8	45.2	33.3	−0.4	0.72
Noise estimate correct trials	0.4	0.1	0.4	0.1	−0.1	0.96
Noise estimate error trials	2.0	1.0	2.0	1.1	−0.3	0.80
CRN amplitude (μV)	2.5	1.9	3.4	2.2	−1.7	0.10
ERN amplitude (μV)	−0.7	2.5	−0.3	1.8	−0.8	0.44
ERN difference amplitude (μV)	−1.8	1.1	−1.6	1.3	0.7	0.47
CRN latency (ms)	64.8	22.9	66.0	30.3	−0.2	0.86
ERN latency (ms)	72.1	15.2	66.2	14.3	1.5	0.14
ERN difference latency (ms)	74.0	14.3	66.4	13.9	2.0	0.05
Correct-trial Pe amplitude (μV)	0.4	1.3	0.4	1.5	0.0	0.97
Error-trial Pe amplitude (μV)	3.9	2.7	5.7	3.1	−2.3	0.03
Pe difference amplitude (μV)	1.8	1.3	2.6	1.3	−2.5	0.02

BDI-II, Beck Depression Inventory—2nd Edition; STAI, State Trait Anxiety Inventory; RT, response time; ERP, event-related potential; CRN, correct-related negativity; ERN, error-related negativity; Pe, post-error positivity; difference, error minus correct.

### Procedure overview

Order of study procedures was identical between the mindfulness and control groups. Participants initially completed a demographics questionnaire and mood measures (e.g., BDI-II, STAI) followed by acquisition of a blood-pressure baseline, placement of the EEG recording net, and completion of either a mindfulness meditation exercise or control listening exercise (described below). Immediately following the mindfulness or control exercise participants completed a modified Eriksen flanker task while EEG and behavioral data were recorded. Two blood pressure readings each 2 min apart were taken at baseline, the end of the mindfulness or control exercise, and at the end of the flanker task. The two readings at each portion were averaged together for statistical analyses to increase reliability. Following the flanker task participants were debriefed about the study aims and provided course credit or $10 per hour for participation.

### Mindfulness and control exercises

Both the mindfulness and control exercises came from Jon Kabat-Zinn's *Mindfulness for Beginners* two-disk CD set (Kabat-Zinn, 2006). Specifically, individuals in the mindfulness group were provided basic instruction on mindfulness meditation and told that they would hear a 14-min audio clip focused on attending to their breathing and being mindful of the moment. They then completed the Mindfulness of Breathing exercise from the *Mindfulness for Beginners* Disk 2 CD (total time = 14:33). Individuals in the control group were provided information about the importance of relaxation and being ethical in our lives. They then listened to two instructional sections from Disk 1 of the *Mindfulness for Beginners* CD entitled Awareness, A Sixth Sense (time = 7:41) and An Ethical Foundation (time = 6:38—total time = 14:19). These tracks only involved educational information on environmental awareness and ethical behaviors and did not involve any meditation practice. Thus, all participants heard the same voice from the same set of CDs for approximately the same amount of time with the only difference being the introduction of a mindfulness exercise in the mindfulness group vs. listening to instruction with no active participation in mindfulness meditation for the control participants.

### Blood pressure data acquisition

Systolic and diastolic blood pressure data were acquired using the oscillmoetric method on a Dinamap Model 8100 automated blood pressure monitor (Critikon Corporation, Tampa, FL, USA). All blood pressure readings were obtained according to manufacturer specifications using a properly sized cuff positioned on the left upper arm. As noted above, two blood pressure readings taken 2 min apart were averaged at baseline, following completion of the mindfulness task, and following completion of the flanker task.

### Modified flanker task

Participants completed a modified version of the Eriksen Flanker Task (Eriksen and Eriksen, 1974). Each trial consisted of either congruent or incongruent arrow stimuli presented in white on a black background of a 17-inch computer monitor approximately 20 inches from the participant's head. Participants were instructed to respond as quickly and accurately as possible with a right-hand key press to the central arrow of a five-arrow array. An index-finger button press was used if the central arrow pointed to the left and a middle-finger button press was used if the central arrow pointed to the right. Flanker stimuli were presented for 100 ms prior to the onset of the central arrow, which remained on the screen for 600 ms. If the participant responded after 1600 ms, the trial was counted as an error of omission. The inter-trial interval varied randomly between 800, 1000, and 1200 ms, with a mean of 1000 ms. All participants completed three blocks of 300 trials (45% congruent; 55% incongruent). Errors of omission were excluded from all data analyses.

### Electroencephalogram recording and reduction

EEG was recorded from 128 scalp sites using a geodesic sensor net and Electrical Geodesics, Inc. (EGI; Eugene, OR) amplifier system (20K nominal gain, bandpass = 0.10–100 Hz). During recording, EEG was referenced to the vertex electrode and digitized continuously at 250 Hz with a 24-bit analog-to-digital converter. Impedances were maintained below 50 kΩ. Data were digitally low-pass filtered at 30 Hz.

Individual-subject response-locked averages were calculated separately for correct-trials and error-trials using a window from −400 ms prior to participant response to 800 ms following participant response. Waveforms were baseline corrected using the 200 ms window from −400 to −200 ms prior to participant response. Eye blinks were removed from the segmented waveforms using independent components analysis (ICA) in the ERP PCA Toolkit (Dien, 2010). The ICA components that correlated at least 0.9 with the scalp topography of two blink templates were removed from the data (Dien et al., 2010). Trials were considered unusable if more than 15% of channels were marked bad. Channels were marked bad if the fast average amplitude exceeded 100 μV or if the differential average amplitude exceeded 50 μV. Data were average re-referenced and used the polar average reference effect (PARE) correction.

**Figure 1:**
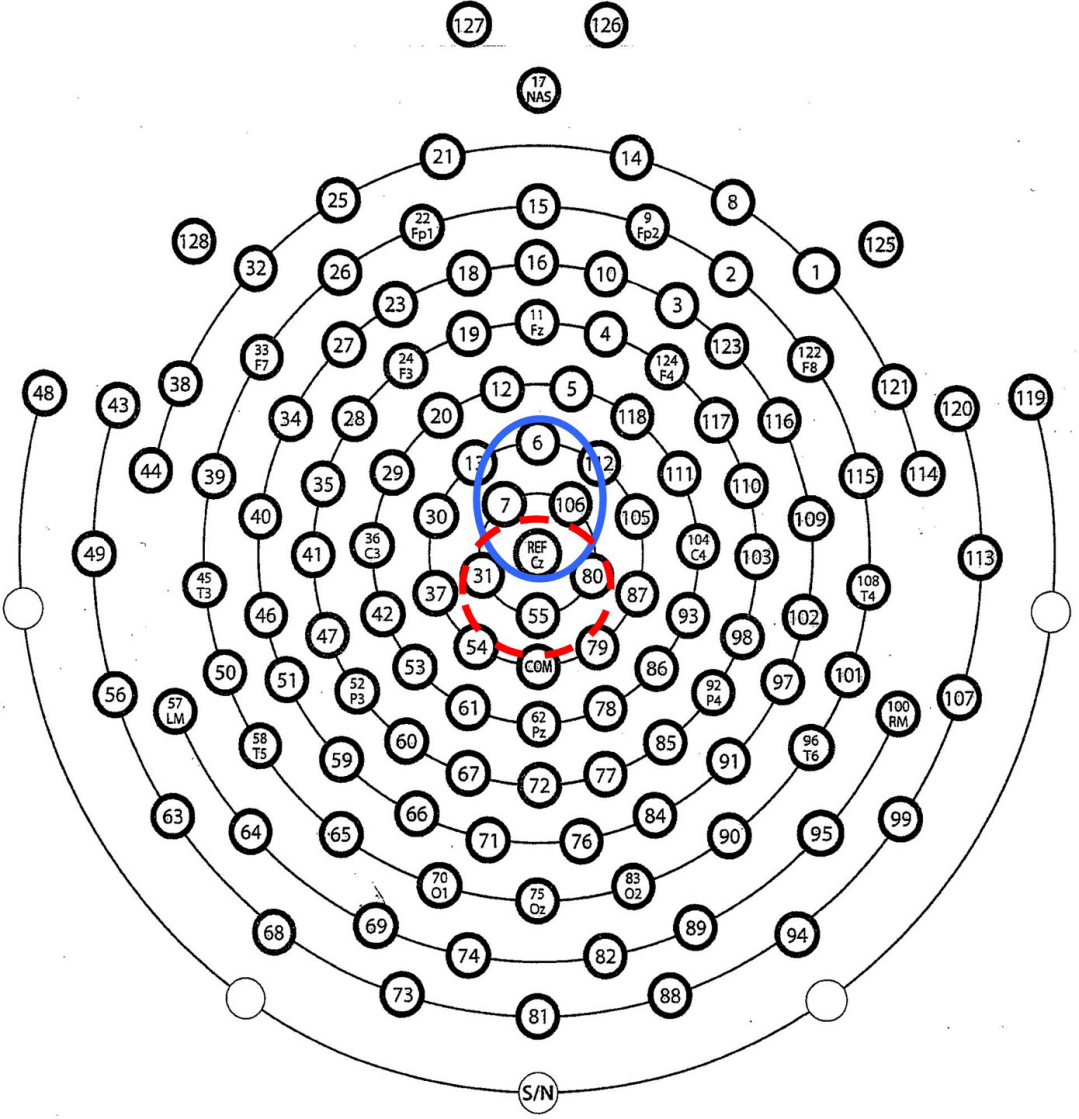
**Sensor layout for the Electrical Geodesics Inc. (EGI) 128-channel hydrocel sensor net**. Electrode locations averaged for measurement of the error-related negativity (ERN) are in the blue circle; the red circle identifies locations for measurement of the post-error positivity (Pe).

**Figure 2:**
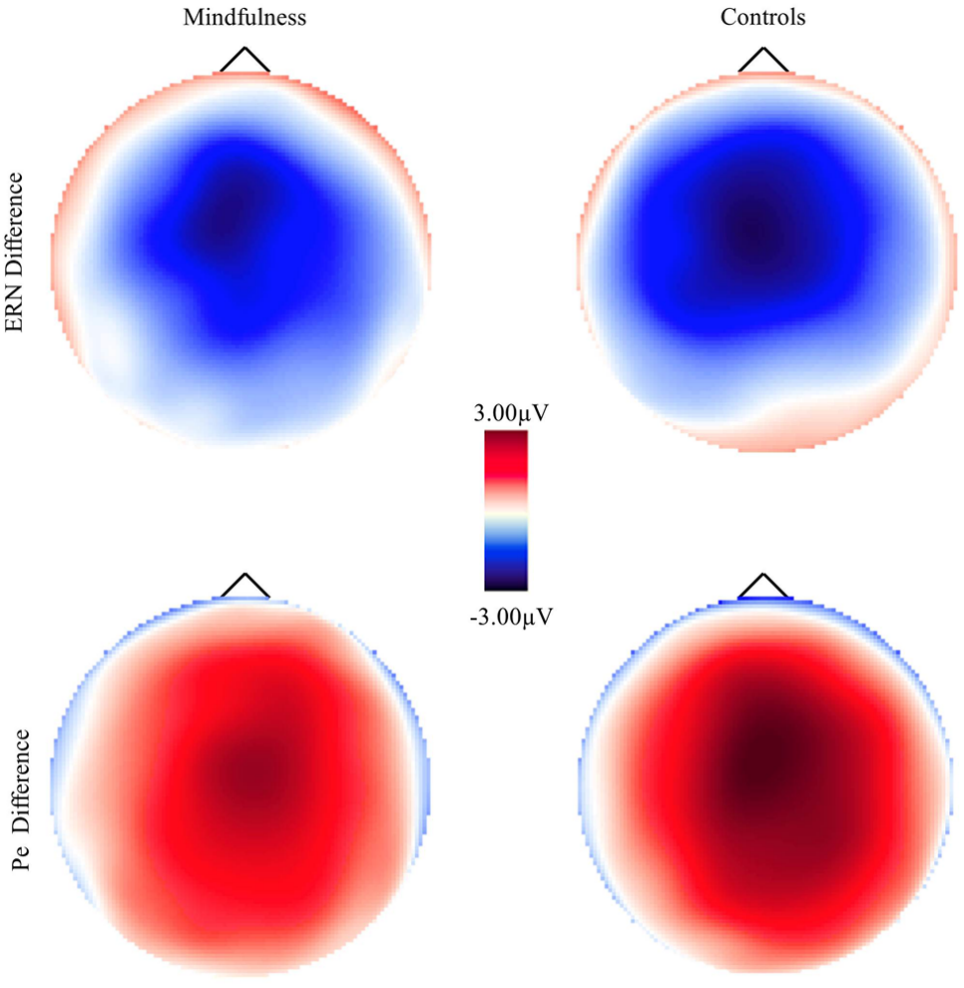
**Scalp voltage maps for the error-minus-correct error-related negativity (ERN) and post-error positivity (Pe) as a function of group**.

Correct-trial and error-trial ERN amplitudes were averaged across four fronto-central electrode sites (numbers 6 [FCz], 7, 106, and Ref [Cz]; see Figure 1 for sensor layout) and extracted as the mean amplitude from 0 to 100 ms following participant response. We used an average across multiple electrodes due to the increased reliability associated with extracting data as the average of multiple electrodes with a similar scalp distribution (see Figure 2) rather than a single electrode (Larson et al., 2010). Latency was extracted as the average time of the most negative peak within the 0–100 ms window averaged across the four frontocentral electrodes. Error-trial and correct-trial Pe amplitudes were extracted as the mean amplitude from 150 to 300 ms post-response at electrode sites 31, 55 [CPz], 80, and Ref [Cz]; see Figure 1). We did not calculate latency of the Pe as it tends to be slower component without a distinct peak and functional significance to latency measurements. Mean amplitudes were chosen for amplitude data extraction because they are more reliable and robust against bias and error due to noise in the ERP data than other ERP extraction techniques such as peak amplitude (Luck, 2005; Clayson et al., 2013).

### Data analysis

Given that several studies show a consistent association between blood pressure and mindfulness meditation (e.g., Carlson et al., 2007; Zeidan et al., 2010; Ankad et al., 2011) we first analyzed the blood pressure data as a manipulation check using 3-Time (baseline, post-mindfulness, post-flanker) × 2-Group (mindfulness, control) repeated measures analyses of variance (ANOVAs) on systolic and diastolic blood pressure values. Mean response time (RT) and error rate data were analyzed using separate 2-Group × 2 Congruency (congruent, incongruent) repeated-measures ANOVAs, whereas electrophysiological and post-error slowing RT data were analyzed using separate 2-Group × 2-Accuracy (correct, error; or in the case of post-error slowing, post-correct and post-error) ANOVAs. We report p*artial-eta*^2^ (η^2^_*p*_) for ANOVA effect sizes and used the Huynh–Feldt epsilon adjustment to correct for possible violations of sphericity for factors with more than two levels. Significant main effects and interactions were decomposed using planned contrasts. Finally, to examine the relationship between physiological variables (i.e., blood pressure) and ERP amplitudes, we conducted separate partial correlations for each group controlling for baseline blood pressure between the post-mindfulness and post-flanker task blood pressure values and ERN amplitude and latency and Pe amplitude. We controlled for baseline blood pressure values to ensure any correlations were due to the variables we manipulated rather than pre-testing blood pressure values.

## Results

### Blood pressure

**Figure 3:**
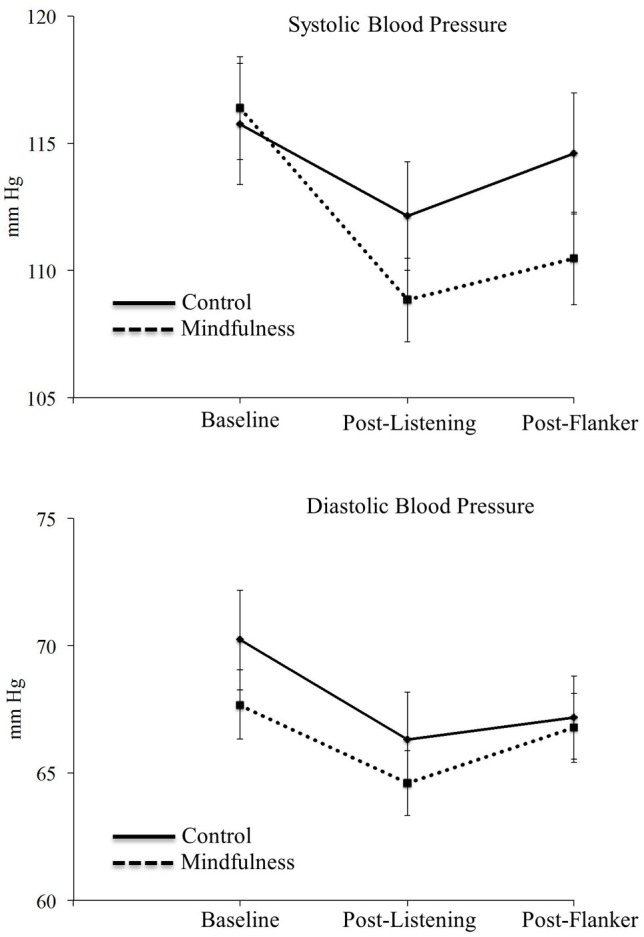
**Mean systolic (top) and diastolic (bottom) blood pressures for at baseline, following the listening exercises (mindfulness or control), and following the flanker task**. Error bars represent the standard error.

We first examined systolic and diastolic blood pressure at baseline, following mindfulness or control exercises, and following the flanker task to ensure that there was a difference in participant response to the mindfulness and control exercises. Systolic and diastolic blood pressure changes as a function of group are presented in Figure 3. The 2-Group × 3-Time ANOVA on systolic blood pressure yielded a significant main effect of time, *F*_(2, 106)_ = 22.12, *p* < 0.001, η^2^_*p*_ = 0.29. Compared to baseline, systolic blood pressure was significantly lower both following the manipulation, *F*_(1, 53)_ = 37.90, *p* < 0.001, η^2^_*p*_ = 0.42, and following the flanker task, *F*_(1, 53)_ = 17.75, *p* < 0.001, η^2^_*p*_ = 0.25; systolic blood pressure was also significantly lower immediately following the manipulation than after the flanker task, *F*_(1, 53)_ = 6.60, *p* = 0.01, η^2^_*p*_ = 0.11. More importantly, the Group × Time interaction was also significant, *F*_(2, 106)_ = 4.42, *p* = 0.01, η^2^_*p*_ = 0.08. Relative to baseline, individuals in the mindfulness group had disproportionately lower systolic blood pressure values following the manipulation, *F*_(1, 53)_ = 4.64, *p* = 0.04, η^2^_*p*_ = 0.08, and following the flanker task, *F*_(1, 53)_ = 7.96, *p* = 0.007, η^2^_*p*_ = 0.13, than controls. There were no significant differences between groups at baseline or between the end of the manipulation and the end of the flanker task, *F*s < 0.26, *p*s > 0.61. The main effect of group was not significant, *F*_(1, 53)_ = 0.67, *p* = 0. 42, η^2^_*p*_ = 0.01. Taken together, results for systolic blood pressure suggest the mindfulness manipulation was successful in disproportionately decreasing blood pressure relative to the control condition and that this effect remained present through the end of the flanker task.

The 2-Group × 3-Time ANOVA on diastolic blood pressure showed a significant main effect of time, *F*_(2, 106)_ = 10.42, *p* < 0.001, η^2^_*p*_ = .16, with decreased diastolic BP both after the manipulation after the flanker task relative to baseline, *F*s > 6.38, *p*s < 0.02, and decreased diastolic BP immediately after the manipulation relative to after the flanker task, *F*_(1, 53)_ = 4.32, *p* = 0.04, η^2^_*p*_ = 0.08. The main effect of group, *F*_(1, 53)_ = 0.57, *p* = 0.45, η^2^_*p*_ = 0.01, and the Group × Time interaction, *F*_(2, 106)_ = 0.99, *p* = 0.37, η^2^_*p*_ = 0.02, were not significant, indicating that there was not a disproportionate effect of the brief mindfulness manipulation on diastolic blood pressure. Overall, however, systolic blood pressure results suggest that even the brief mindfulness manipulation in novice participants produced a physiologic effect that remained over the course of the flanker task.

### Behavioral data

Mean RT and error rate data as a function of group are presented in Table 1. The Group × Congruency ANOVA on RTs showed the expected main effect of congruency with longer RTs on incongruent trials relative to congruent trials, *F*_(1, 53)_ = 1342.28, *p* < 0.001, η^2^_*p*_ = 0.96. The Group × Congruency interaction, *F*_(1, 53)_ = 1.41, *p* = 0.24, η^2^_*p*_ = 0.03, and the main effect of group, *F*_(1, 53)_ = 0.01, *p* = 0.93, η^2^_*p*_ < 0.01, were not statistically significant. For post-error slowing, a Group × Accuracy (post-error, post-correct) ANOVA showed **c**onsistent post-error slowing when collapsed across groups as seen by a significant main effect of accuracy, *F*_(1, 53)_ = 27.85, *p* < 0.001, η^2^_*p*_ = 0.34, with slower RTs following errors than accurate responses (see Table 1). The Group × Accuracy interaction, *F*_(1, 53)_ = 0.04, *p* = 0.84, η^2^_*p*_ = 0.001, and the main effect of group, *F*_(1, 53)_ = 0.001, *p* = 0.99, η^2^_*p*_ = 0.001, were non-significant.

Findings were similar for error rates. The expected main effect of congruency with increased errors on incongruent trials relative to congruent trials was present, *F*_(1, 53)_ = 1342.28, *p* <.001. The Group × Congruency interaction, *F*_(1, 53)_ = 1.41, *p* = 0.24, η^2^_*p*_ = 0.03, and the main effect of group, *F*_(1, 53)_ = 0.01, *p* = 0.93, η^2^_*p*_ < 0.01, were not statistically significant.

### Event-related potential data

**Figure 4:**
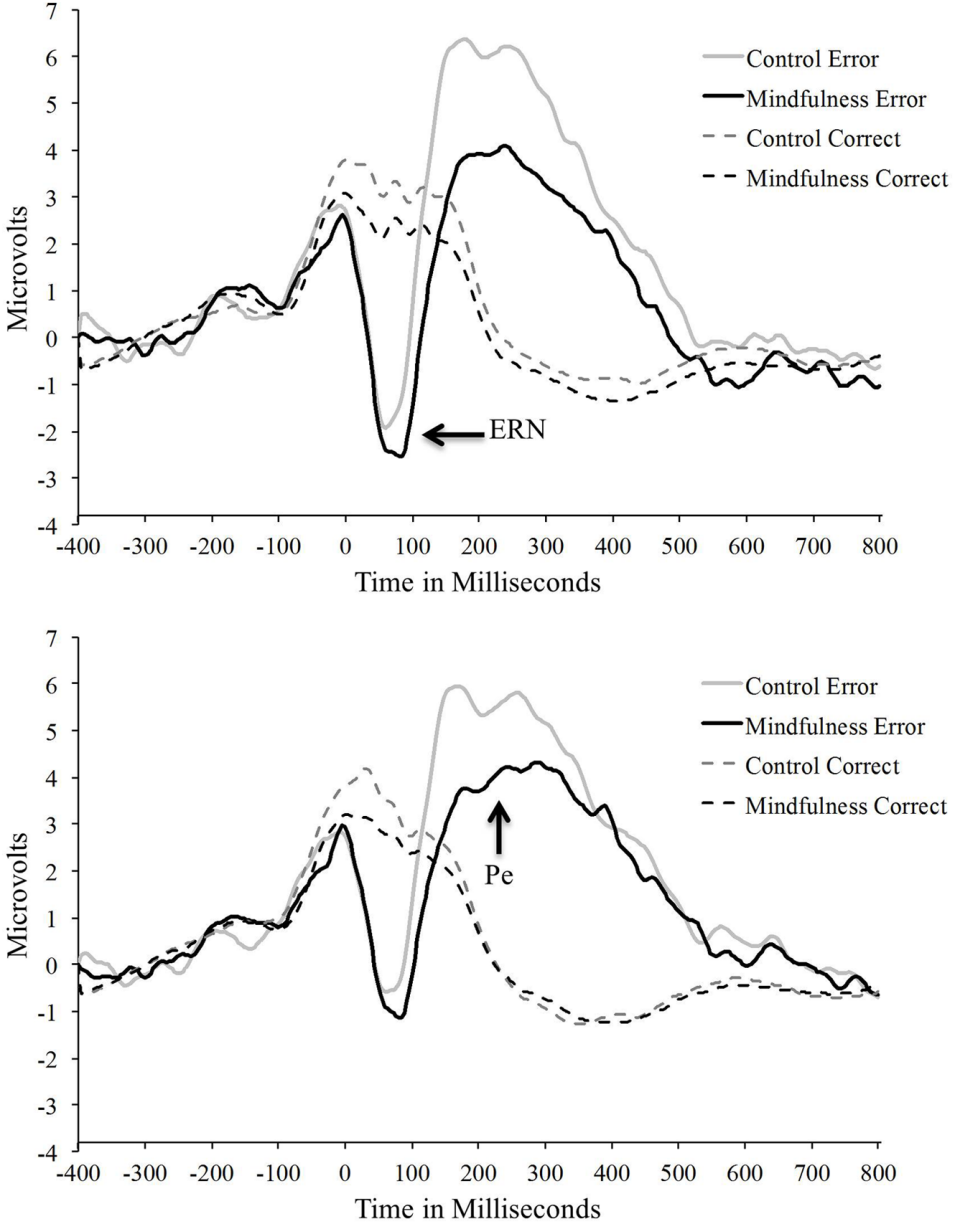
**Grand average waveforms as a function of group representing the error-related negativity (ERN; top) averaged across frontocentral electrode locations and the post-error positivity (Pe; bottom) averaged across central electrode locations**.

Electrophysiological data, including number of trials per condition, noise estimates, and mean ERP amplitudes and latencies, are presented in Table 1. Grand averaged ERN and Pe waveforms for both mindfulness and control groups are presented in Figure 4 with corresponding error-minus-correct difference voltage maps in Figure 2. Groups did not significantly differ in number of trials retained for averaging in the ERPs, *F*s < 0.52, *p*s > 0.47, and had similar noise estimates between groups, *F*s < 0.01, *p*s > 0.94 (Schimmel, 1967). Thus, signal-to-noise ratio does not appear to have significantly affected the ERP results.

The Group × Accuracy ANOVA on ERN amplitude showed a significant main effect of accuracy, *F*_(1, 53)_ = 106.74, *p* < 0.001, η^2^_*p*_ = 0.67, with the expected more negative ERN on error trials than correct trials. The Group × Accuracy interaction was not statistically significant, *F*_(1, 53)_ = 0.51, *p* = 0.948, η^2^_*p*_ = 0.01, nor was the main effect of group, *F*_(1, 53)_ = 2.19, *p* = 0.15, η^2^_*p*_ = 0.04. Findings indicate no group-related differences in ERN amplitude. Similarly, the Group × Accuracy ANOVA on ERN latency showed non-significant findings for all main effects and interactions, *F*s < 1.03, *p*s > 0.31, suggesting that latency was not influenced by accuracy or mindfulness group.

Analysis of the Pe revealed larger Pe amplitudes to error trials than correct trials, *F*_(1, 53)_ = 150.37, *p* < 0.001, η^2^_*p*_ = 0.74. More importantly, there was a significant Group × Accuracy interaction, *F*_(1, 53)_ = 6.20, *p* = 0.02, η^2^_*p*_ = 0.11. Groups significantly differed on error-trial Pe amplitude with individuals in the control group showing increased Pe amplitude than those in the mindfulness group; there were no group differences for correct-trial Pe amplitude (see Table 1, Figure 4). The main effect of group was non-significant, *F*_(1, 53)_ = 3.29, *p* = 0.08, η^2^_*p*_ = 0.06. Taken together, Pe amplitudes significantly differentiated groups, with the differences being primarily due to increased Pe amplitude for controls on error trials.

### Correlational analyses

Partial correlations controlling for baseline blood pressure values between blood pressure following the listening tasks (mindfulness or control) and following the flanker task and ERP values for control and mindfulness participants are presented in Table 2. There were no significant correlations for either group.

**Table 2:** **Partial correlations controlling for baseline blood pressure between blood pressure and ERPs**.

	**Listening systolic**	**Flanker systolic**	**Listening diastolic**	**Flanker diastolic**
Control ERN amplitude	0.15 (0.48)	0.16 (0.46)	−0.07 (0.75)	0.14 (0.52)
Control ERN latency	0.26 (0.22)	0.23 (0.27)	0.20 (0.35)	−0.39 (0.06)
Control Pe amplitude	0.16 (0.43)	0.12 (0.58)	−0.15 (0.48)	−0.13 (0.54)
Mindfulness ERN amplitude	−0.25 (0.22)	−0.11 (0.61)	−0.28 (0.16)	−0.19 (0.36)
Mindfulness ERN latency	0.31 (0.12)	0.28 (0.17)	−0.05 (0.81)	0.13 (0.53)
Mindfulness Pe amplitude	−0.03 (0.88)	−0.36 (0.07)	−0.33 (0.10)	−0.18 (0.39)

Partial correlations are presented as r-value (p-value). Listening refers to the measurements following the listening to either mindfulness or control audio.

## Discussion

Previous research has documented a variety of meditation interventions and the effects such interventions have on cognitive performance and cardiovascular functioning. We examined the influence of a brief mindfulness intervention on behavioral and electrophysiological indices of cognitive control and performance monitoring—specifically the ERN and Pe components of the scalp-recorded ERP and RTs, post-error slowing, and error rates. The mindfulness manipulation was effective as it successfully altered participant physiology as evidenced by disproportionately decreased systolic blood pressure in the mindfulness participants relative to controls both after the manipulation and through the end of the flanker task. Behaviorally, however, there were no significant differences between individuals in the mindfulness and control conditions for RTs, post-error slowing, or error rates. Similarly, there were no differences in ERN amplitude or latency. There was, however, a significant difference between groups for amplitude of the Pe. These differences were due to significant between-groups differences in Pe amplitude for error trials, but not correct trials. Thus, our findings primarily indicate that a brief mindfulness intervention can decrease autonomic physiology and amplitude of a performance monitoring ERP, but does not influence performance-related indices of cognitive control such as RTs or error rates.

One of the key findings from this study is that a very brief mindfulness meditation intervention was associated with a considerable decrease in systolic blood pressure in the mindfulness participants relative to controls. This effect can be largely attributed to the mindfulness intervention as the control participants heard the same voice, from the same CDs, for a similar amount of time compared to the mindfulness participants. Thus, the primary difference between groups was that the control heard about the importance of relaxation and living ethically whereas the mindfulness group actually engaged in mindfulness meditation. Zeidan et al. (2010) observed similar changes in blood pressure after a 2-week meditation training among meditators compared to a sham and control group; however, the authors concluded that these group differences were inconclusive due to a lack of evidence linking changes in blood pressure to the interventions employed. These authors further suggested that such changes in blood pressure are only noticed after brief training only when people are assigned to a stress-inducing condition. Our findings, in contrast, suggest that a stress-inducing task and long-term mindfulness intervention is not necessary for blood pressure change. The mindfulness intervention was approximately 15 min long and was associated with a considerable decrease in systolic blood pressure in the absence of a pre-task stressor. Indeed, our experimental design provided considerable control allowing us to make a strong case that brief meditation was responsible for altering systolic blood pressure. Replication is needed both with and without stress-inducing tasks, but it appears that one 15-min session of brief mindfulness training appears sufficient to at least temporarily reduce blood pressure in non-meditators.

There were also significant between-groups differences for the amplitude of the Pe component of the ERP, with less positive mean Pe amplitude in the mindfulness group. As noted above, amplitude of the Pe is generally associated with conscious processing or awareness of errors or the motivational significance/salience of an error (Nieuwenhuis et al., 2001; Overbeek et al., 2005; Endrass et al., 2007, 2012; Larson and Perlstein, 2009; Shalgi et al., 2009; Ullsperger et al., 2010; Hughes and Yeung, 2011). A possible interpretation of this finding may be that individuals who complete a brief mindfulness intervention are less aware of their errors or find their errors less attentionally-engaging or motivationally-salient. One additional possibility of considerable interest is that the Pe may reflect an orienting response toward errors that is related to autonomic physiology (Ullsperger et al., 2010; Wessel et al., 2011). Given previous findings indicating that the orienting response to errors and the amplitude of the Pe are related to autonomic nervous system response (e.g., Hajcak et al., 2003; Wessel et al., 2011), it seems quite possible that mindfulness meditation reduces both indices of acute autonomic nervous system arousal such as systolic blood pressure and more phasic measures such as Pe amplitude. The absence of a significant correlation between blood pressure and Pe values, however, decreases the clarity of this possibility.

The functional significance of the Pe component of the ERP needs further elucidation. Similarly, the finding of Pe-related differences associated with mindfulness meditation requires further study, particularly because our Pe results stand in contrast to the absence of group differences in Pe amplitude in the Teper and Inzlicht (2013) study in experienced meditators. It is possible that there is a difference between a brief and fleeting exposure to meditation state and long-term meditation traits (such as emotional acceptance as measured in the Teper and Inzlicht study). That said, studies tend to indicate improved attention, rather than decreased attention, in those who practice mindfulness meditation (Jha et al., 2007; Tang et al., 2007; Zeidan et al., 2010; Moore et al., 2012) and there are several studies that do not show affective trait-related changes in Pe amplitude (Clayson et al., 2012; Larson et al., 2013; Pfabigan et al., 2013). Thus, while an interesting finding, future studies need to directly manipulate error salience, error awareness, orienting to errors, and mindfulness meditation in the moment vs. as a more ingrained trait to clarify the Pe and mindfulness relationship.

In contrast to the reduction in systolic blood pressure that persisted through the end of the flanker task and the reduction in error-related Pe amplitude, there were no significant differences between groups for behavioral performance or ERN amplitudes or latencies. These findings were contrary to our hypotheses and previous research that indicates mindfulness and acceptance are associated with increased-amplitude ERN (Teper and Inzlicht, 2013). Possible reasons for the general absence of differences between groups in RTs, error rates, and the ERN may be in the acuity of the intervention and the naivety of the participants. Studies comparing expert and novice meditators during resting and meditative states found that expert meditators showed more activation than their novice counterparts in brain regions associated with attention, particularly the ACC, during functional MRI studies of meditation (Lazar et al., 2000; Brefczynski-Lewis et al., 2007; Holzel et al., 2007). The only other study of performance monitoring electrophysiology in mindfulness meditation (Teper and Inzlicht, 2013) used a participant sample of expert meditators with at least 1 year of meditation experience and an average of 3.19 years experience. Thus, it is possible that a cumulative effect of frequent meditation and acquiring some expert skill is required to significantly impact behavioral and ERP reflections of performance monitoring.

Given the possibility that more exposure and practice is needed to truly understand the relationship between cognitive control and mindfulness, several studies show desirable changes in attention and cognitive control abilities following brief meditation interventions (Jha et al., 2007; Tang et al., 2007; Zeidan et al., 2010; Moore et al., 2012). Previous work shows that as few as 3 h of mind-body meditation training are needed to induce reliable changes in ACC functioning (Tang et al., 2007, 2009); however, up to 11 h of training is needed to achieve changes in neural indices of white matter integrity (Tang et al., 2010). The trend in the current data is consistent with that of Teper and Inzlicht (2013) showing increased ERN amplitude in mindful participants relative to controls. Future studies comparing indices of performance monitoring before and after longer-term meditation in a dose dependent fashion are needed to determine the relative amounts of meditation training needed to induce neural or performance changes. Such studies could clearly inform the question of the amount of meditation needed to influence subsequent cognitive performance.

Our findings, particularly those for the ERN, have implications for recent studies looking to determine whether amplitude of the ERN is related to trait personality variables or state-related changes in affect (Olvet and Hajcak, 2009, 2012; Clayson et al., 2012; Larson et al., 2013). Several studies show that brief changes in affect (either positive/congruent affect or negative/incongruent affect) will alter ERN amplitude (Larson et al., 2006; Wiswede et al., 2009; Inzlicht and Tullettt, 2010; Boksem et al., 2011; van Wouwe et al., 2011; Inzlicht and Al-Khindi, 2012). One recent study that used a pre-test to post-test design and orthogonally manipulated mood valence and arousal using a music mood induction technique showed that state-related valence and arousal did not significantly affect ERN amplitude, the error-trial minus correct-trial amplitude difference was associated with arousal ratings (higher arousal associated with increased difference) but not state-related valence (Larson et al., 2013). Other studies suggest no state-related changes in ERN amplitude (e.g., Moser et al., 2005; Clayson et al., 2012) or state-related changes that are moderated by more trait-related personality characteristics (Olvet and Hajcak, 2012). The current results indicate that, despite considerable changes in systolic blood pressure suggesting a more relaxed mood state, ERN amplitude is not affected by state change in mindfulness. These findings need future augmentation by data directly measuring state-related mood changes associated with the meditation; however, it stands to reason that the changes in ERN amplitude observed among expert meditators may reflect a trait-related variables that developed through hundreds of hours of meditation practice or from a personality more disposed to participating in meditation practices.

Limitations of the study should be considered. First, the experimental control achieved directly manipulating the brief mindfulness and control interventions decrease the external validity of the study—particularly given that all participants had never participated in any form of previous meditation. Furthermore, it is possible that the control participants experienced some positive effects of listening to the recording of thoughts on environmental awareness and ethical behaviors as they may relate to mindfulness practices. Indeed, the absence of between-groups findings for the ERN or behavioral performance may be due to the similarity of the control and experimental manipulations. Second, we did not directly measure subjective ratings of mindfulness during the course of the task, choosing instead to rely on a more objective index of blood pressure to determine if the mindfulness intervention was effective in differentiating groups. Third, null results are often difficult to interpret as potential outside factors, such as changes in affective state over time, were not measured. It is possible that extraneous factors masked true differences between groups. Finally, because we had started data collection before the previous study of ERN amplitudes and mindfulness was published (Teper and Inzlicht, 2013) we did not measure some important variables, such as emotional acceptance, that appear to mediate/moderate the relationship between ERN amplitudes and mindfulness.

In summary, our results suggest that a very brief mindfulness intervention in non-meditators decreases systolic blood pressure and is associated with reduced amplitude of the Pe ERP over the short term, but does not immediately influence ERN amplitude or behavioral indices of cognitive control. Future research using graded levels of mindfulness experience and paradigms that directly measure error awareness would provide a necessary step to understanding the relationship between performance monitoring and mindfulness meditation.

### Conflict of interest statement

The authors declare that the research was conducted in the absence of any commercial or financial relationships that could be construed as a potential conflict of interest.
